# Alpha-2A Adrenoceptor Agonist Guanfacine Restores Diuretic Efficiency in Experimental Cirrhotic Ascites: Comparison with Clonidine

**DOI:** 10.1371/journal.pone.0158486

**Published:** 2016-07-06

**Authors:** Giovanni Sansoè, Manuela Aragno, Raffaella Mastrocola, Giulio Mengozzi, Maurizio Parola

**Affiliations:** 1 Division of Gastroenterology, Gradenigo Hospital, Torino, Italy; 2 Department of Clinical and Biological Sciences, University of Torino, Torino, Italy; 3 Clinical Biochemistry Laboratory, San Giovanni Battista Hospital, Torino, Italy; Taipei Veterans General Hospital, TAIWAN

## Abstract

**Background:**

In human cirrhosis, adrenergic hyperfunction causes proximal tubular fluid retention and contributes to diuretic-resistant ascites, and clonidine, a sympatholytic drug, improves natriuresis in difficult-to-treat ascites.

**Aim:**

To compare clonidine (aspecific α_2_-adrenoceptor agonist) to SSP-002021R (prodrug of guanfacine, specific α_2A_-receptor agonist), both associated with diuretics, in experimental cirrhotic ascites.

**Methods and Results:**

Six groups of 12 rats were studied: controls (G1); controls receiving furosemide and potassium canrenoate (G2); rats with ascitic cirrhosis due to 14-week CCl_4_ treatment (G3); cirrhotic rats treated (over the 11^th^-14^th^ CCl_4_ weeks) with furosemide and canrenoate (G4), furosemide, canrenoate and clonidine (G5), or diuretics and SSP002021R (G6). Three rats of each group had their hormonal status and renal function assessed at the end of 11^th^, 12^th^, 13^th^, and 14^th^ weeks of respective treatments.Cirrhotic rats in G3 and G4 gained weight over the 12^th^-14^th^ CCl_4_ weeks. In G4, brief increase in sodium excretion over the 11^th^-12^th^ weeks preceded worsening of inulin clearance and natriuresis (diuretic resistance). In comparison with G4, the addition of clonidine (G5) or guanfacine (G6) to diuretics improved, respectively, sodium excretion over the 11^th^-12^th^ CCl_4_ weeks, or GFR and electrolytes excretion over the 13^th^-14^th^ CCl_4_ weeks. Natriuretic responses in G5 and G6 were accompanied by reduced catecholamine serum levels.

**Conclusions:**

α_2A_-receptor agonists restore glomerular filtration rate and natriuresis, and delay diuretic-resistant ascites in experimental advanced cirrhosis. Clonidine ameliorates diuretic-dependent natriuresis just for a short time.

## Introduction

Refractory ascites, which occurs in most cirrhotic patients with end-stage liver disease and avid sodium retention, comprises diuretic-intractable and diuretic-resistant ascites [1]. In order to treat the latter, which is genuinely unresponsive to the use of diuretics, several therapies have been tested, including repeated paracentesis with intravenous albumin, peritoneal venous shunt, and transjugular intrahepatic portosystemic shunt (TIPS), but none of these procedures was found to improve survival in patients with advanced liver cirrhosis [2, 3]. TIPS may even precipitate hepatic failure and worsen survival rate in patients with high Child-Pugh score [4].

The mechanisms of diuretic-resistant ascites, whose essential clinical features are progressive increase in body weight and decrease in natriuresis despite doses of diuretics that were previously achieving a diuresis, are manifold: peripheral arterial vasodilatation, portal hypertension, reduction of the effective arterial blood volume, and permanent activation of endogenous anti-natriuretic and renal vasoconstrictor mechanisms (renin-angiotensin [RAS] and sympathetic nervous [SNS] systems, non-osmotic hypersecretion of vasopressin [ADH]) [5, 6]. Diuretic-resistant ascites occurs as a result of extreme vascular underfilling with maximal activation of these anti-natriuretic systems [1]. Specifically, the activated SNS and RAS stimulate kidney arterial vasoconstriction, which eventually leads to decrease in renal blood flow and glomerular filtration rate. Additionally, norepinephrine and angiotensin II increase reabsorption of sodium in the proximal renal tubule, which leads to negligible response to diuretics and to enhanced secretion of renin [7].

In patients with cirrhosis and diuretic-resistant ascites, clonidine, an α_2_-adrenoceptor agonist that decreases central sympathetic outflow, release of norepinephrine from vascular neuroeffector junctions [8] and portal pressure [9], has been tried as adjunct to common diuretics with promising results. Clonidine, associated with the α_1_-adrenoceptor agonist midodrine and standard medical therapy (SMT), was superior to SMT alone in the control of ascites in cirrhotic patients [10]. Moreover, clonidine improved the effects of diuretics (spironolactone alone or the combination of furosemide and spironolactone) in patients with advanced liver cirrhosis and ascites [11, 12].

Clonidine’s binding affinity does not differ appreciably among the many α_2_-receptors so far described. Indeed, five α_2_-receptors subtypes exist: α_2A_, α_2B1,_ α_2B2,_ α_2C,_ and α_2D_, which belong to the group A of rhodopsin-like G protein-coupled receptor class [13]. Clonidine systemic effects do not depend preferentially on stimulation of α_2_-receptors that are pre- or post-junctional (i.e. pre- or post-synaptic), located either in the central nervous system, in the wall of peripheral blood vessels, or in the kidney [13, 14].

The similar affinity of clonidine for this multitude of α_2_-adrenoceptors is a manifest drawback and may render the results of clonidine itself sub-optimal in the treatment of the ascitic patients that are ill-responsive to diuretics. Indeed, clonidine, through stimulation of endothelial α_2D_-receptors, may enhance vascular production of nitric oxide (NO), leading to arterial vasodilatation, hypotension [14] and worsening of the hyperdynamic circulation of cirrhotic patients. Furthermore, enhanced NO synthesis increases the expression of apical Na^+^-K^+^-2Cl^-^ cotransporters in the loop of Henle, and therefore renal sodium retention [15]. Finally, stimulation of α_2B_-adrenoceptors, located in the basolateral membrane of the proximal renal tubule [16], leads to accelerated sodium reabsorption even in this nephron segment [16, 17].

Guanfacine, a different α_2_-adrenoceptor agonist, has approximately 60-fold more selectivity for α_2A_-receptors than clonidine [18] and does not lower arterial blood pressure significantly in patients with arterial hypertension [19]. Guanfacine, through specific stimulation of renal α_2A_-adrenoceptors, increases osmolar clearance and sodium excretion in a peculiar naltrexone (opioid receptor antagonist)-sensitive manner, according to the established natriuretic function of renal α_2A_-adrenoceptors [18]. The rat kidney contains α_2A_-receptors, which are located in the inner stripe of the renal cortex and in the outer medulla [16, 20, 21], where stimulation of these post-synaptic receptors antagonizes the antidiuretic effects of ADH [22]. Indeed, guanfacine leads to significant aquaretic effects even in experimental ascitic cirrhosis [23]. For these reasons, guanfacine may represent a much better candidate drug, among α_2_-receptor agonists, than clonidine in order to blunt adrenergic hyperfunction and restore diuretic efficiency in advanced cirrhotic ascites.

With this premise in mind, the present study has been designed to achieve two major aims: a) to characterize, in terms of renal function and hormonal status, the occurrence and identify the timing of unresponsiveness to traditional diuretic therapy in the experimental model of rats with advanced carbon tetrachloride (CCl_4_)-dependent cirrhosis; b) to investigate in such a stage of experimental decompensated cirrhosis the hypothesized advantage of guanfacine over clonidine, when these drugs are added to diuretics in order to restore their natriuretic efficiency.

## Materials and Methods

Studies were performed on 48 male adult Wistar rats with advanced liver cirrhosis and 24 male adult Wistar control rats. All Wistar rats were provided by Harlan Italy, Udine, Italy. Both cirrhotic and control groups were fed with standardized chow and water. Cirrhosis was induced by CCl_4_ (Riedel-de Haën, Sigma-Aldrich, Seelze, Germany) administered by gavage twice weekly for 14 weeks [24]. The pathophysiological progression of this experimental model is highly predictable and reproducible: after 9 weeks, micronodular cirrhosis is evident, rats are devoid of ascites (as assessed by laparotomy) and portal pressure is increased to about 10 mmHg; after 11 weeks, rats present ascites and their mean portal pressure is 24 mmHg; after 14 weeks cirrhotic rats develop renal failure and eventually die [25, 26]. Control rats were studied after 14 weeks of standardized diet. Rats were cared for in compliance with the European Council directives (No. 86/609/EEC) and with the Principles of Laboratory Animal Care (NIH no. 85–23, revised in 1985). This scientific project was approved by the Ethical Committee of the University of Torino (permit number: D.M. 92/2010-B). In this study, the following active drugs were administered to the rats according to the protocol described in the next paragraphs: furosemide, Henle’s loop diuretic (Sanofi-Aventis, Milano, Italy); potassium canrenoate, aldosterone receptor antagonist (Teofarma, Pavia, Italy); clonidine, α_2_-adrenoceptor agonist (Boehringer Ingelheim, Milano, Italy). Finally, SSP-002021R, oral prodrug of guanfacine, selective α_2A_-adrenoceptor agonist, was provided by Shire, Basingstoke, U.K.

### Animal groups

Furosemide, canrenoate, clonidine, and SSP-002021R were dissolved in distilled water to obtain different solutions to be administered orally to the rats in 400 μl of fluid. The animals were divided into six groups of twelve rats: controls receiving no intervention (group G1); controls receiving three times a week for 4 weeks oral furosemide (0.5 mg/Kg b.w.) and oral potassium canrenoate (2 mg/Kg b.w.) (G2); rats with ascitic cirrhosis due to 14-week CCl_4_ administration and receiving no active drug (G3); cirrhotic rats treated with oral furosemide (0.5 mg/Kg b.w. three times a week) plus oral potassium canrenoate (2 mg/Kg b.w. three times a week) between the beginning of the 11^th^ and the end of the 14^th^ week of CCl_4_ (G4); cirrhotic rats treated with oral furosemide, oral potassium canrenoate (see above dosage), and oral clonidine (0.3 mcg three times a week) between the beginning of the 11^th^ and the end of the 14^th^ week of CCl_4_ (G5); cirrhotic rats treated with oral furosemide, oral canrenoate, and the oral prodrug of guanfacine (SSP002021R, selective α_2A_-adrenoceptor agonist, 5 mg/kg b.w. three times a week) between the beginning of the 11^th^ and the end of the 14^th^ week of CCl_4_ (G6). Dosage of furosemide and potassium canrenoate was patterned on respective standard daily human dosage. A dosage of clonidine 0.3 mcg on alternate days was chosen: previous experiments in this laboratory showed clonidine 0.5 mcg caused arterial hypotension in cirrhotic rats and blunted further the effects of diuretics (unpublished data), and published papers showed the effectiveness of low, non-hypotensive doses of clonidine (75 mcg once or twice daily in adult human cirrhotic patients) [10–12]. The dosage of SSP002021R used in this study was established by the provider of the drug (Shire, Basingstoke, U.K.)

### Study protocol

Rats belonging to G1–G6 were weighed, studied and finally sacrificed, three at a time, at the end of weeks 11, 12, 13, and 14 of observation or CCl_4_ administration, with or without the above active treatments. All rats treated with active drugs were studied within 8 hours after the latest drug administration. Each day of study, rats were anesthetized with a mixture of Ketavet 100 (Farmaceutici Gellini, Sabaudia, Italy) and Rompum (Xylazine, Bayer A.G., Leverkusen, Germany) (4:1 v:v) by intraperitoneal injection (0.5 ml mixture/200 g b. wt.) [27]; laparotomy was performed and the urinary bladder was emptied before clamping the urethral orifice for further urine collection. Shortly thereafter, inulin (IN) 10% (w/v) (Laevosan-Gesellschaft, Linz/Donau, Austria) plus para-aminohippurate (PAH) 20% (w/v) (Nephrotest, BAG Gmbh, Munich, Germany) were administered into the caudal vein as a priming bolus followed by a continuous infusion, in order to assess glomerular filtration rate (GFR) and renal plasma flow (RPF) by means of their respective steady-state plasma clearances (CIN and CPAH) [28, 29]. When 90 minutes of IN and PAH infusion had elapsed (i.e. once their steady-state plasma concentrations had been reached), cardiac blood was sampled to assess plasma osmolality and concentrations of inulin, PAH, sodium, and potassium. Blood samples withdrawn at this time were also used to measure plasma renin activity (PRA) and concentrations of vasopressin (ADH), aldosterone (A), epinephrine (E), and norepinephrine (N). Finally, urinary bladder was emptied to collect the urine volume produced during the 90 min of IN and PAH venous infusion. This urine was used to determine its osmolality and the excretion of sodium and potassium. Rats were then killed by exsanguination through the aorta. Anesthetized rats in each group had their mean arterial pressure evaluated through tail sphygmomanometry, as described elsewhere [25], before performing laparotomy.

### Plasma and urine analyses

Plasma and urinary concentrations of electrolytes and IN and PAH plasma concentrations were measured as described elsewhere [26, 30, 31]. Plasma A, ADH, N, E, and PRA were determined according to standard procedures [25, 32].

### Calculations

Sodium and potassium clearances (CNa and CK) were calculated through the usual formula [32]. Inulin clearance (CIN) and para-aminohippurate clearance (CPAH) were calculated through the steady-state plasma clearance formula as:

Cx=Infusion rate (x)/ssP-x

where ssP-x is the steady-state plasma concentration of x. CIN and CPAH were taken as measures of GFR and RPF, respectively [28, 29]. Filtration fraction (FF) and filtered sodium load (FlNa) were calculated through the usual formulae [32].

Fractional sodium excretion (FENa) and fractional potassium excretion (FEK) were also calculated [27].

Tubular free-water reabsorption (TF-WR) was calculated, following Rose and Post [33], through the formula:

TF-WR=Cosm–V

where V is the urinary output (ml/min) and Cosm is the osmolar clearance, which was computed via the usual formula:

Cosm=(Uosm×V)/Posm

where Uosm and Posm are urine and plasma osmolarities, respectively.

Mean arterial pressure (MAP) was calculated from the formula:

1/3(systolic blood pressure–diastolic blood pressure)+diastolic blood pressure

### Statistical analysis

Statistical comparisons of renal function or hormone levels in rats belonging to different G1–G6 groups, performed after definite times of exposure to CCl_4_, were made by one-way analysis of variance (ANOVA) followed by Tukey’s LSD post-hoc comparisons. Comparisons among rats belonging to the same group, but studied at different times (i.e. measurements of mean ± SD of weeks 11–12 vs. weeks 13–14 of observation or CCl4) were made through one-tailed Wilcoxon rank sum test for unpaired data. All results are expressed as means ± SD. Significance is accepted at the 5% probability level.

## Results

### Identification of unresponsiveness to diuretics in rats with experimental ascitic cirrhosis

**Fig 1:**
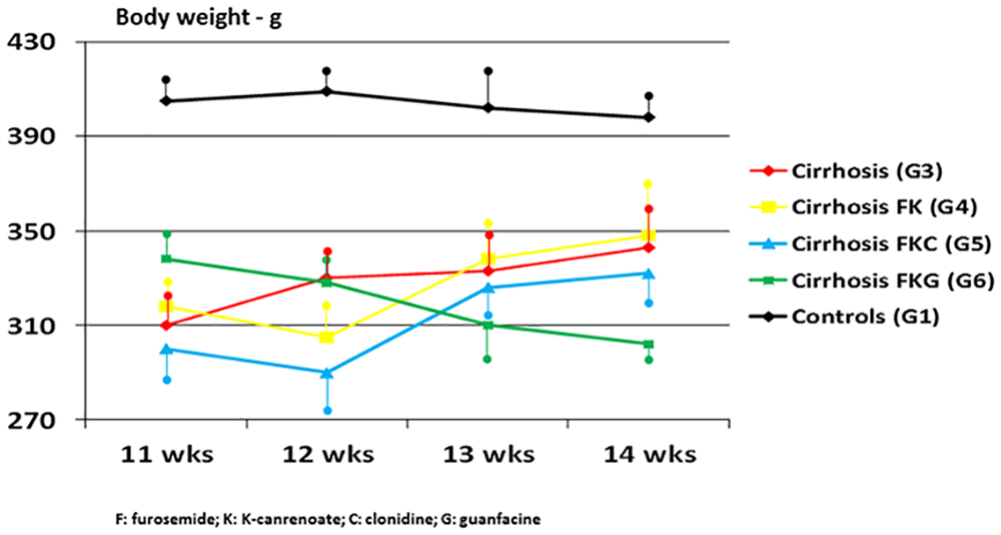
Progressive weight gain of untreated cirrhotic rats (G3, red line) and of cirrhotic rats treated with diuretics (G4, yellow line) or with diuretics plus clonidine (G5, blue line). Further groups depicted: G1 (healthy controls, black line), G6 (cirrhotic rats treated with diuretics plus oral prodrug of guanfacine, green line). Mean measurements ± SD of three rats studied at a time in each group are depicted.

**Fig 2:**
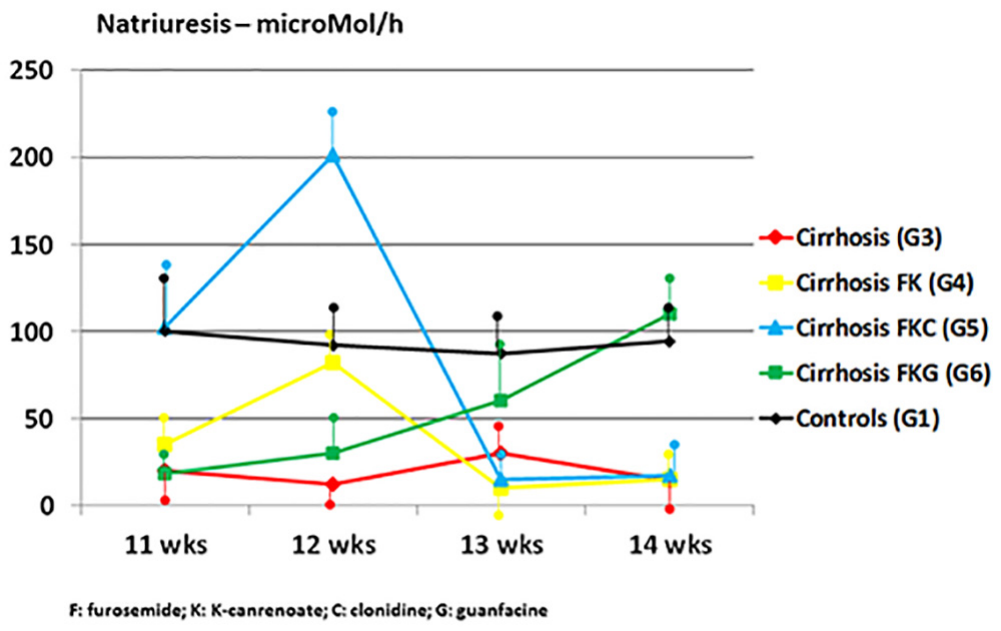
Transient natriuretic effects in G4 (cirrhotic rats treated with furosemide and potassium canrenoate, yellow line) and G5 (cirrhotic rats treated with diuretics plus clonidine, blue line) over CCl_4_ weeks 11–12. Progressive natriuretic effects in G6 (cirrhotic rats treated with diuretics plus oral prodrug of guanfacine, green line). Further groups depicted: G1 (healthy controls, black line), G3 (untreated cirrhotic rats, red line). Mean measurements ± SD of three rats studied at a time in each group are depicted.

**Fig 3:**
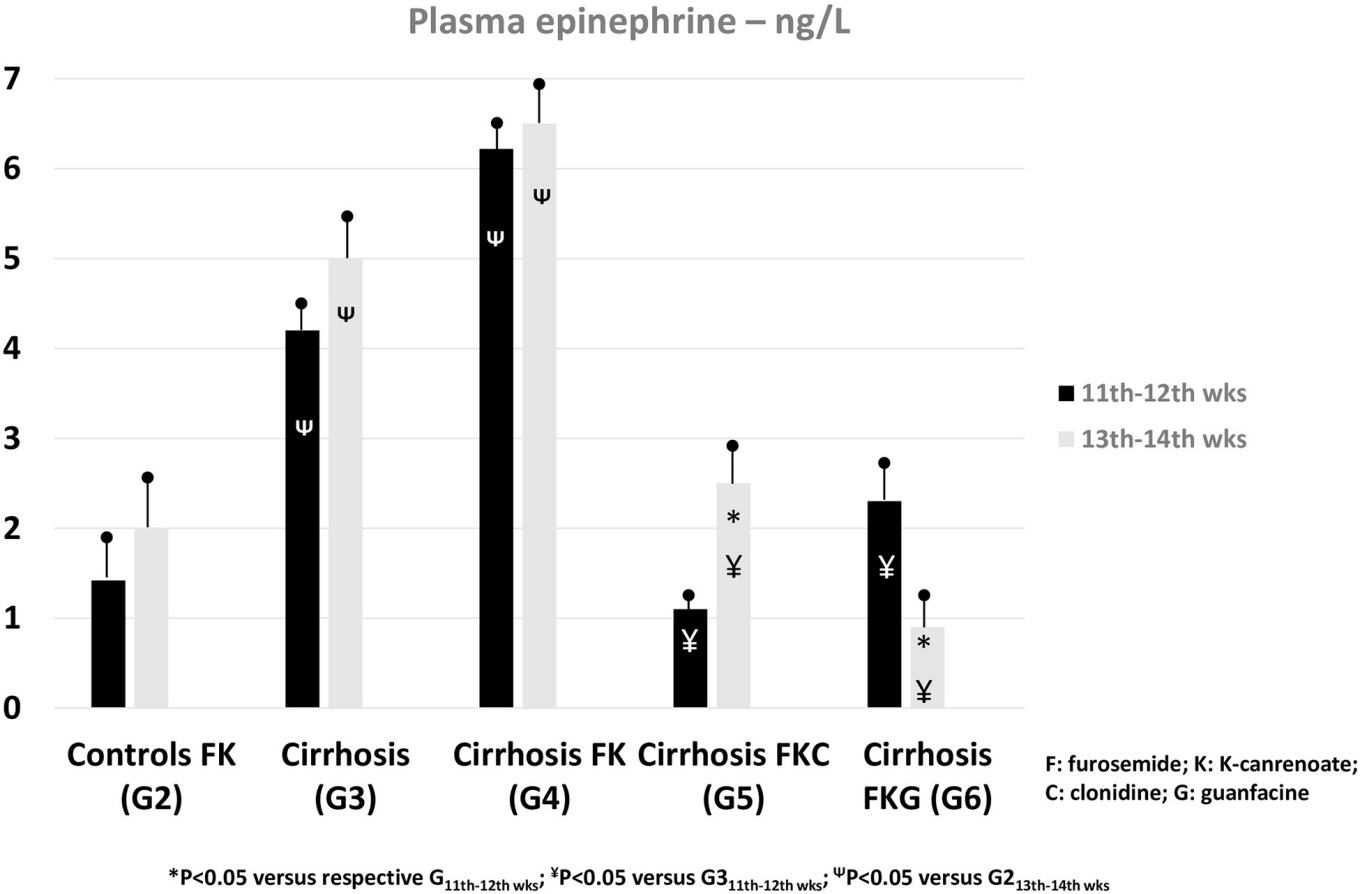
Graphical depiction of adrenergic hypertone in cirrhotic rats, treated (G4) or not (G3) with diuretics. Early (G5) and late (G6) blunting of adrenergic function in cirrhotic rats receiving, respectively, clonidine or guanfacine, along with diuretics.

Normal rats (G2) had a progressive increase in urine volume and sodium excretion without derangement of renal function over the four weeks of diuretic treatment (Table 1). The body weight of G3 and G4 cirrhotic rats increased progressively over the 12th-14th weeks of CCl_4_ due to ascites accumulation, irrespective of standard dosage of diuretics in G4 (Table 1, Fig 1). From the end of week 12 onwards, in G4 the occurrence of unresponsiveness to diuretics was characterized by values of GFR, renal plasma flow, sodium excretion even lower (Table 1), and systemic catecholamine levels even higher than in cirrhotic rats not treated with diuretics (G3) (Figs 2 and 3). This means that overt diuretic-resistant ascites in this model of advanced cirrhosis occurs at the end of 12 weeks of CCl_4_.

**Table 1:** Renal function. Comparisons between means ± SD of GFR, RPF, urine volume, urine sodium excretion rate, etc. taken on weeks 11–12 (Group GX_A_) vs. weeks 13–14 (Group GX_B_) or among different G1–G6 groups. In each group, worsening of clinical parameters underlined, improvements in **bold** print (weeks 13–14, Group GX_B_, vs. weeks 11–12, Group GX_A_).

	Body weight (g)	CIN (ml/min)	CPAH (ml/min)	FF (%)	Urine volume (ml/h)	Natriuresis (εmol/h)
Group G1_A_	407 ± 53	2.1 ± 0.19	4.5 ± 0.8	46 ± 6	0.72 ± 0.07	94 ± 14
G1_B_	400 ± 46	2.3 ± 0.26	4.07 ± 0.81	56 ±10	0.69 ± 0.07	91 ± 18
G2_A_	401± 48	2 ± 0.13	4.13 ± 0.73	48 ± 8	**0.69 ± 0.08**	**94 ± 12**
G2_B_	377 ± 36	2.15 ± 0.2	4.6 ± 0.93	46 ± 6	0.91 ± 0.09*	121 ± 12*
G3_A_	320 ± 18	1.68 ± 0.13	3.6 ± 0.64	52 ± 7	0.325 ± 0.04	19.5 ± 5
G3_B_	340 ± 12*	1.44 ± 0.13	2.5 ± 0.21*	50 ± 7	0.315 ± 0.04*	21 ± 3
G4_A_	312 ± 11	1.7 ± 0.12	3.2 ± 0.45	56 ± 8	0.54 ± 0.06	65 ± 6^Ψ^
G4_B_	340 ± 12*	1.2 ± 0.11*^Ψ^	2.1 ± 0.14*^Ψ^	57 ±7	0.28 ± 0.02*	15.5 ± 2*^Ψ^
G5_A_	295 ± 7	2.0 ± 0.12^¥^	3.9 ± 0.18^¥^	74 ± 8	1.015 ± 0.06^¥^	157.5 ± 16^¥^
G5_B_	328 ± 7*	1.2 ± 0.21*	3.57 ±0.85	47 ± 6*	0.205 ± 0.03*	18 ± 3*
G6_A_	**333 ± 12**	**1.5 ± 0.11**	**3.61 ± 0.25**	**38 ± 5**	**0.34 ± 0.06**	**17.5 ± 6**
G6_B_	306 ± 11*^Ψ^	2.1 ± 0.19*^Ψ^	4.01 ± 0.21*^Ψ^	52 ± 6*	0.90 ± 0.07*^Ψ^	85 ± 14*^Ψ^

Data are means ± SD.

*P<0.05 versus respective GX_A_;

^Ψ^P<0.05 versus G3_B_;

^¥^P<0.05 versus G4_A_.

Statistical comparisons of renal function or hormone levels in rats belonging to different G1–G6 groups, performed after definite times of exposure to CCl_4_, were made by one-way analysis of variance (ANOVA) followed by Tukey’s LSD post-hoc comparisons. Comparisons among rats belonging to the same group, but studied at different times (i.e. measurements of mean ± SD of weeks 11–12 vs. weeks 13–14 of observation or CCl4) were made through one-tailed Wilcoxon rank sum test for unpaired data.

CIN: steady-state plasma clearance of inulin; CPAH: steady-state plasma clearance of para-aminohippurate; FF: filtration fraction.

### Renal function

In cirrhotic rats, the favourable effects of clonidine, when added to furosemide and canrenoate (G5), was prompt and resulted in an increase in urine flow rate and sodium excretion that paralleled and expanded the slight one due to diuretics only in G4 (Table 1, Fig 2). Nonetheless, the advantage of treatment with clonidine was also transient and followed by accelerated derangement of renal function over weeks 13–14 (Tables 1 and 2; Fig 2). Guanfacine was slower that clonidine in achieving an increase in diuresis and natriuresis, but its effects were progressive and significant mostly on weeks 13–14 of CCl_4_ (Table 1, Fig 2). Actually, guanfacine, added to diuretics, caused a progressive improvement in renal function (GFR and RPF) over the four weeks of observation (weeks 11–14) (Table 1). Guanfacine, when added to the traditional diuretic treatment of ascites over weeks 11–14, caused a steady and progressive increase in urinary potassium excretion rate (Table 2). Guanfacine, at the used dosage and associated with diuretics, did not affect significantly tubular free-water reabsorption. Notably, in cirrhotic rats treated with diuretics alone (G4) or with diuretics plus clonidine (G5), severe GFR deterioration occurred during the 13^th^ and 14^th^ weeks of CCl_4_ (Table 1). In these two groups, which had their sodium excretion rates transiently increased over the first two weeks of respective treatment, different mechanisms might have led to similar anti-natriuretic effects in the long run: clonidine promptly increases GFR and natriuresis (Table 1 and Fig 2), which are then followed by later deterioration of renal function; diuretics cause an immediate tubular diuretic effect that leads to decrease in effective arterial blood volume and earlier deterioration of GFR due to urinary fluid loss not counterbalanced by any sympatholytic action (Tables 1 and 3; Figs 2 and 3).

**Table 2:** Renal function. Comparisons between means ± SD of FENa, kaliuresis, plasma Na, etc. taken on weeks 11–12 (Group GX_A_) vs. weeks 13–14 (Group GX_B_) or among different G1–G6 groups. In each group, worsening of clinical parameters underlined, improvements in **bold** print (weeks 13–14, Group GX_B_, vs. weeks 11–12, Group GX_A_).

	FENa (%)	Kaliuresis εmol/h)	Plasma Na (mEq/l)	Plasma K (mEq/l)	Cosm (ml/h)	TF-WR (ml/h)
Group G1_A_	2.2 ± 0.13	31 ± 5	141 ± 2	4.1 ± 0.6	1.9 ± 0.22	1.18 ± 0.14
G1_B_	2.1 ± 0.12	34 ± 6	139 ± 4	3.8 ± 0.8	1.92 ± 0.21	1.23 ± 0.22
G2_A_	**2.2 ± 0.2**	47 ± 6	141 ± 3	4.4 ± 1.1	1.93 ± 0.27	1.24 ± 0.25
G2_B_	3.5 ± 0.22*	47 ± 8	137 ± 2	3.6 ± 2.1	2.02 ± 0.51	1.11 ± 0.11
G3_A_	1.6 ± 0.27	47.5 ± 9	135 ± 3	3.5 ± 1.4	1.47 ± 0.33	1.15 ± 0.20
G3_B_	1.2 ± 0.19	40 ± 14	136 ± 2	3.2 ± 0.8	1.01 ± .036	0.7 ± 0.21
G4_A_	2.1 ± 0.47	80 ± 16	137 ± 4	3.6 ± 1.1	1.77 ± 0.33	1.2 ± 0.22
G4_B_	0.7± 0.19*^Ψ^	40 ± 10*	132 ± 3	3.1 ± 0.9	1.01 ± 0.26*	0.7 ± 0.26
G5_A_	2.3 ± 0.4	90 ± 17	139 ± 3	3.1 ± 0.8	2.56 ± 0.72	**1.75 ± 0.38**
G5_B_	1.1 ± 0.29*	15 ± 7*	135 ± 3	3.8 ± 0.9	0.455 ± 0.06*	0.25 ± ± 0.06*
G6_A_	**1.3 ± 0.32**	**50 ± 14**	141 ± 2	3.7 ± 0.4	**1.04 ± 0.27**	0.7 ± 0.2
G6_B_	2.8 ± 0.6*^Ψ^	97 ± 19*^Ψ^	136 ± 2*	3.0 ± 0.3*	1.85 ± 0.4*	0.95 ± 0.22

Data are means ± SD.

*P<0.05 versus respective GX_A_;

^Ψ^P<0.05 versus G3_B_.

Statistical comparisons of renal function or hormone levels in rats belonging to different G1–G6 groups, performed after definite times of exposure to CCl_4_, were made by one-way analysis of variance (ANOVA) followed by Tukey’s LSD post-hoc comparisons. Comparisons among rats belonging to the same group, but studied at different times (i.e. measurements of mean ± SD of weeks 11–12 vs. weeks 13–14 of observation or CCl4) were made through one-tailed Wilcoxon rank sum test for unpaired data.

Cosm: osmolar clearance; FENa: fractional sodium excretion; TF-WR, tubular free-water reabsorption.

**Table 3:** Hormonal status. Comparisons between means ± SD of PRA, plasma aldosterone, etc. taken on weeks 11–12 (Group GX_A_) vs. weeks 13–14 (Group GX_B_) or among different G1–G6 groups. In each group, worsening of clinical parameters underlined, improvements in **bold** print (weeks 13–14, Group GX_B_, vs. weeks 11–12, Group GX_A_).

	PRA (ng/ml/h)	Plasma A (pg/ml)	Plasma N (ng/l)	Plasma E (ng/l)	Plasma ADH (pg/ml)
Group G1_A_	4.2 ± 0.7	420 ± 73	131 ± 18	0.98 ± 0.8	246 ± 27
G1_B_	5.0 ± 1.2	388 ± 87	98 ± 20	1.31 ± 0.2	221 ± 26
G2_A_	4.8 ± 1.4	499 ± 66	199 ± 20	1.42 ± 0.21	244 ± 50
G2_B_	5.22 ± 1.2	545 ± 64	322 ± 50*	1.99 ± 0.14	199 ± 38
G3_A_	9.2 ± 1	1602 ± 131	593 ± 66	4.2 ± 0.51	245 ± 79
G3_B_	20.0 ± 2.6*	2003 ± 167*	738 ± 80*	5.0 ± 0.32	368 ± 36*
G4_A_	11.22 ± 0.6^¥^	2121 ± 145^¥^	832 ± 87^¥^	6.22 ± 0.67^¥^	300 ± 46
G4_B_	19.9 ± 2.1*	2560 ± 131*^Ψ^	1002 ± 85*^Ψ^	6.50 ± 0.87	400 ± 45*
G5_A_	8.7 ± 0.8	1122 ± 88	200 ± 36^¥^	1.1 ± 0.21^¥^	333 ± 67
G5_B_	9.2 ± 0.6	1245 ± 81	242 ± 30^¥^	2.5 ± 0.48*^¥^	401 ± 130
G6_A_	8.8 ± 0.9	1006 ± 53	**190 ± 22**^¥^	**2.3 ± 0.45**^¥^	287 ± 53
G6_B_	9.0 ± 0.8^Ψ^	1187 ± 52^Ψ^	67 ± 14*^¥^	0.9 ± 0.14*^¥^	345 ± 40

Data are means ± SD.

*P<0.05 versus respective GX_A_;

^Ψ^P<0.05 versus G3_B_;

^¥^P<0.05 versus G3_A_.

Statistical comparisons of renal function or hormone levels in rats belonging to different G1–G6 groups, performed after definite times of exposure to CCl_4_, were made by one-way analysis of variance (ANOVA) followed by Tukey’s LSD post-hoc comparisons. Comparisons among rats belonging to the same group, but studied at different times (i.e. measurements of mean ± SD of weeks 11–12 vs. weeks 13–14 of observation or CCl4) were made through one-tailed Wilcoxon rank sum test for unpaired data.

A, aldosterone; ADH, vasopressin; E, epinephrine; N, norepinephrine; PRA, plasma renin activity.

### Hormonal status (Table 3)

**Fig 4:**
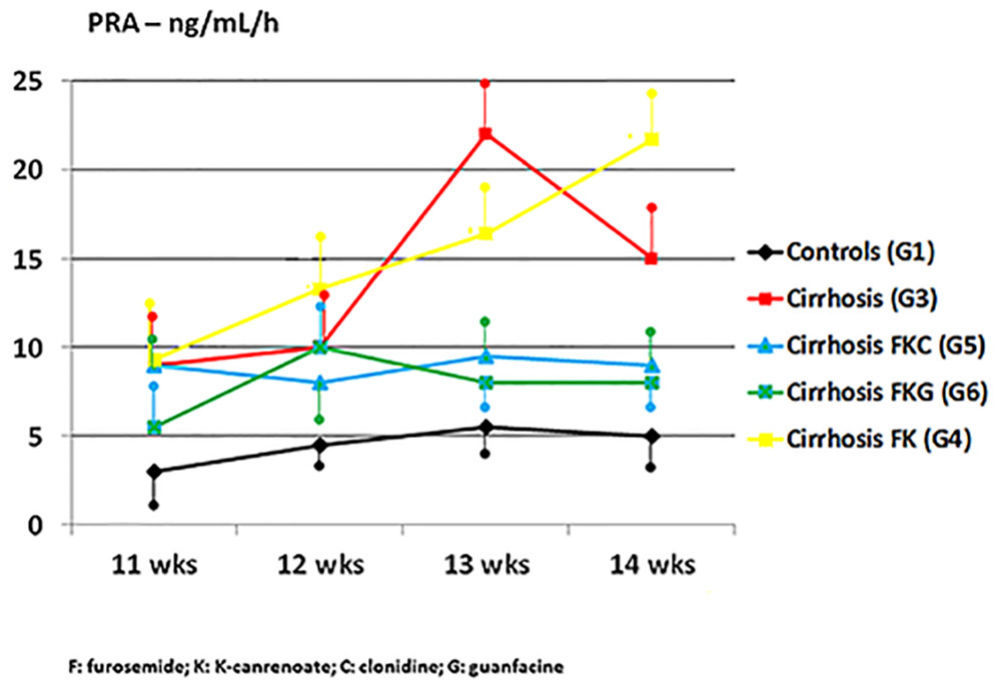
Marked increase in PRA (concurrent with development of diuretic-unresponsive ascites) over CCL_4_ weeks 13–14 in cirrhotic rats (G3, untreated, red line, and G4, receiving diuretics only, yellow line). Blunting of renin secretion in G5 (cirrhotic rats treated with diuretics plus clonidine, blue line) and G6 (cirrhotic rats treated with diuretics plus oral prodrug of guanfacine, green line). Further group depicted: G1 (untreated controls, black line). Mean measurements ± SD of three rats studied at a time in each group are depicted.

α_2_-adrenergic agonists blunted the adrenergic hyperfunction that characterizes advanced liver cirrhosis, as shown by reduced levels of serum norepinephrine in ascitic cirrhotic rats treated with clonidine (G5) or guanfacine (G6) plus diuretics compared to cirrhotic rats untreated (G3) or treated with sole diuretics (G4). When the behaviour of epinephrine serum levels is considered (Fig 3), blunting of adrenergic function was early and transient with clonidine and, instead, progressive and long-lasting with guanfacine. The effect of guanfacine on adrenergic function contributes to the increased natriuretic effects and the amelioration of GFR described previously. In the advanced stage of liver disease, cirrhotic rats (G3) showed a progressive and severe hyper-reninism (i.e. secondary aldosteronism), which was maximal during overt refractory ascites (i.e. on CCl_4_ weeks 13 and 14) (Fig 4). As expected, even this phenomenon was positively affected by the blunting of adrenergic function caused by α_2_-agonists (Fig 3).

### Mean arterial pressure

When compared to absolute controls (G1), significantly lower values of MAP were measured in the cirrhotic group treated with sole diuretics (G4) over CCl_4_ weeks 12–14 (all P<0.05) (data not shown in tables).

## Discussion

This study identifies the timing of overt unresponsiveness to diuretics in the standard model of experimental ascitic cirrhosis due to chronic CCl_4_ administration [34]. This unresponsiveness to diuretics occurs at the end of 12 weeks of CCl_4_, when two weeks of successful treatment with furosemide and potassium canrenoate have elapsed.

Our assertion of the occurrence of such a resistance to diuretics is not, and could not be, based on the accepted definition of refractory ascites, as this is diagnosed in patients with advanced liver cirrhosis [35]. In our cirrhotic rats diuretic-unresponsive ascites occurs after 12 weeks of CCl_4_ because of the manifest lack of effects of traditional diuretics—effects we accurately measured once a week and not daily—and because of the progressive weight gain despite the diuretics (Table 1 and Figs 1 and 2). Diuretic-resistant ascites is preceded by significant diuretic responses to the association of furosemide and anti-aldosterone drugs, and by increased adrenergic function and secondary aldosteronism during such a diuretic response (Tables 1 and 3, Figs 2, 3 and 4). Harbinger of diuretic-resistant ascites, which occurs over weeks 13–14 of CCl_4_, i.e. after two weeks of diuretics, is a further increase in secondary aldosteronism, representing a critical loss of effective arterial blood volume (Fig 4). This closely mimics the occurrence of human diuretic-resistant ascites previously responsive to diuretics. Of course, since we were not interested in the diagnosis of diuretic-intractable ascites in our experimental model, we did not monitor the extra-renal side effects of diuretics (e.g. possible signs of hepatic encephalopathy) that characterize diuretic-intractable ascites in cirrhotic patients [35].

Guanfacine, selective α_2A_-adrenoceptor agonist, when added to the traditional diuretic treatment of ascites, apparently prevents the occurrence of diuretic-resistant ascites, at least over the length of our study (i.e. 14 weeks of CCl_4_) (Fig 2). On the contrary, clonidine, aspecific α_2_-adrenoceptor agonist and sympatholytic agent, just amplifies the diuretic effects of furosemide and potassium canrenoate in the weeks before the occurrence of diuretic resistance (Fig 2).

Notably, guanfacine, a sympatholytic agent itself, in our cirrhotic rats caused a later attenuation of catecholamine release, as assessed by the serial measurement of epinephrine and norepinephrine plasma levels (Table 3, Fig 3). This is associated with blunting of renin production (Fig 4) and progressive recovery of GFR over CCl_4_ weeks 13–14 (Table 1) These hormonal and renal effects are accompanied by progressive increase in urinary excretions of sodium and potassium, which are maximal after 4 weeks of guanfacine (i.e. on CCl_4_ week 14) (Tables 1, 2 and Fig 2). The concurrent increase in urinary excretion of sodium and potassium (Tables 1 and 2) may suggest some guanfacine-dependent increase in delivery of tubular fluid to the loop of Henle, where furosemide exerts its natriuretic and kaliuretic action.

The natriuretic effect of clonidine, as adjunct to diuretics, is maximal during CCl_4_ weeks 11 and 12, is accompanied by transient improvement of renal function and decrease in catecholamine release, but is followed by severe deterioration of GFR over the following weeks of treatment (Tables 1 and 3; Figs 2 and 3). This is different from the ephemeral increase in urine flow and sodium excretion rate due to diuretics only, which apparently prompts early and uncontrolled stimulation of adrenergic function and secondary aldosteronism (Tables 1 and 3; Figs 3 and 4).

Clonidine’s and guanfacine’s beneficial effects, transient the former and later the latter, may have the following reasons. First, clonidine exerts its adrenolytic effects earlier than the oral prodrug of guanfacine (Table 3, Fig 3) because of the need of metabolic activation of the latter, metabolic activation that might be slowed down in a setting of liver failure. Second, aspecific α_2-_adrenergic stimulation by clonidine may elicit some potential anti-natriuretic forces that are the consequence of stimulation of NO synthesis [14–17]. Third and last, it was demonstrated that α_2A_-receptor stimulation (by guanfacine or analogues) leads to direct tubular diuretic effects [18].

So far, only clonidine has been tested in order to ameliorate the effects of diuretics in patients with advanced cirrhosis and ascites. And these attempts were successful [10– 12]. It is conceivable that, in patients with decompensated cirrhosis, as well as in our model of experimental ascites, beneficial diuretic effects of clonidine might be short-lived. And, in this probable case, oral prodrug of guanfacine would be worth being tried instead of clonidine, once suitable guanfacine’s dosage and schedule is clearly established in patients with decompensated cirrhosis.

In conclusion, this study represents the first successful use of α_2A_-adrenoceptor agonists in order to increase the diuretic effects of furosemide and anti-aldosterone drugs in the setting of experimental advanced cirrhosis with ascites. No previous clinical or experimental study has ever been performed with this aim. Moreover, we described a suitable experimental model of diuretic-resistant ascites in cirrhotic rats. This model may be useful for further pathophysiological or pharmacological studies. Future efforts to arrange dosage and administration schedule of oral prodrugs of guanfacine for the treatment of cirrhotic patients with ascites seem now worthwhile.

## Abbreviations

A: aldosterone
ADH: vasopressin
CCl_4_: carbon tetrachloride
CIN: steady-state plasma clearance of inulin
CK: potassium clearance
CNa: sodium clearance
Cosm: osmolar clearance
CPAH: steady-state plasma clearance of para-aminohippurate
E: epinephrine
FEK: fractional excretion of potassium
FENa: fractional sodium excretion
FF: filtration fraction
FlNa: filtered sodium load
GFR: glomerular filtration rate
IN: inulin
MAP: mean arterial pressure
N: norepinephrine
NO: nitric oxide
PAH: para-aminohippurate
Posm: plasma osmolality
PRA: plasma renin activity
RAS: renin-angiotensin system
RPF: renal plasma flow
SD: standard deviation
SMT: standard medical therapy
SNS: sympathetic nervous system
TF-WR: tubular free-water reabsorption
TIPS: transjugular intrahepatic portosystemic shunt
Uosm: urine osmolality
